# Management, socioeconomics, and One Health determinants of tick infestation in communal cattle production systems of South Africa

**DOI:** 10.3389/fvets.2026.1806890

**Published:** 2026-05-22

**Authors:** Makhado P. Sedina, Emmanuel O. Njoga, James W. Oguttu

**Affiliations:** 1 Department of Agriculture and Animal Health, College of Agriculture and Environmental Sciences, University of South Africa (UNISA), Roodepoort, South Africa; 2 Department of Veterinary Public Health and Preventive Medicine, Faculty of Veterinary Medicine, University of Nigeria, Nsukka (UNN), Nsukka, Nigeria

**Keywords:** communal cattle production system, economic losses, South Africa, tick control, tick infestation

## Abstract

**Background:**

Tick infestation constrains livestock productivity and predisposes to zoonotic tick-borne disease. Therefore, this study examined management, socio-economic, and the human and environmental determinants of tick infestation in communal cattle production systems in rural South Africa.

**Methods:**

A cross-sectional study was conducted among 168 cattle-owning households across 17 villages in South Africa. Structured and validated questionnaire was used to collect data on socio-demographics, tick control practices, knowledge of tick-borne diseases, and the availability and functionality of cattle-rearing infrastructure. Data were analyzed using descriptive statistics and generalized linear mixed-effects models.

**Results:**

Most households (82.7%) relied on communal grazing and had small herd sizes (median = 7 cattle). Although 95.8% of respondents recognized ticks as disease vectors, only 54.8% practiced regular tick control. Common tick control methods included pour-on acaricides (61.3%) and hand spraying (51.2%). Acaricide rotation was limited (27.4%). Dipping infrastructure was largely inadequate, with 94.6% of the respondents saying that their dip tanks were either non-functional or partially functional. Ninety-eight (58.3%) cattle-owning households were located more than 5 km from a functional facility. Knowledge of tick-borne diseases (3.6%) and zoonotic risks (8.9%) was low, despite frequent human-tick contact (78%). Dysfunctional dipping infrastructure (β = 0.41, *p* < 0.001), low household income (β = 0.29, *p* < 0.001), communal grazing (β = 0.25, *p* = 0.002), and irregular acaricide use (β = 0.33, *p* < 0.001) were significant factors associated with high herd-level tick burden. Majority of farmers (61.9%) spent over ZAR 1,000 (≈USD 62) annually on tick control, with higher tick burdens significantly associated (*p* < 0.01) with reduced cattle market value and delayed sales. Tick infestation was also linked with poor body condition (66.7%), reduced draft power (58.3%), hides damage (51.8%), and increased acaricide expenditure (61.9%).

**Conclusion:**

Persistent tick infestation in communal cattle systems is strongly influenced by socioeconomic constraints, dysfunctional dipping infrastructure, communal grazing, and irregular acaricide use. These factors were associated with substantial self-reported economic burden and elevate human exposure risks. Restoring accessible dipping services, strengthening extension support, and adopting integrated One Health tick control strategies are essential for sustainable tick control in the study area.

## 1 Introduction

Ticks are among the most economically important ectoparasites affecting cattle worldwide, particularly in tropical and subtropical regions where climatic conditions favor their survival and proliferation (1, 2). In sub-Saharan Africa, tick infestations (3–6), as well as pathogenic and antimicrobial resistant bacterial infections (7–14) are major constraints to livestock productivity. Ticks cause direct effects like blood loss, hide damage, reduced draft power, dermatitis and poor body condition (15–17), as well as indirect effects especially transmission of tick-borne diseases (18, 19). In communal cattle systems, where livestock play central roles in food security, income generation, and socio-cultural practices, the impact of ticks extends beyond animal health to household livelihoods and rural economic stability (20).

Communal cattle production systems are characterized by shared grazing resources, unrestricted animal movement, and limited biosecurity (21), creating ecological conditions conducive for tick persistence and transmission. Unlike commercial farming systems, communal cattle production systems often lack structured pasture management, regular veterinary oversight, and functional tick control infrastructure (22–24). These structural limitations could promote continuous tick–host–environment interactions and contribute to the maintenance of endemic tick populations. Evidence from southern Africa consistently shows higher tick burdens and greater exposure to tick-borne diseases in communal systems compared to commercial farms (25–27), underscoring the need for context-specific control strategies.

Tick infestation also represents an underappreciated public health and zoonotic concerns. Some tick species commonly infesting cattle are vectors of zoonotic pathogens, including *Rickettsia, Anaplasma*, and other emerging agents of human disease (28). In communal settings, close human–animal contact, manual tick removal, shared living environments, and limited use of personal protective measures increase the likelihood of human exposure (29). Despite this, tick-borne zoonoses remain poorly recognized in many rural communities, and human health considerations are rarely integrated into livestock-focused tick control programs, reflecting a critical gap in One Health implementation.

Effective tick control in communal systems is further constrained by socio-economic and institutional challenges. Poverty, limited access to acaricides, irregular veterinary extension services, and widespread deterioration of dipping infrastructure undermine the sustainability of traditional control measures. The collapse or dysfunction of communal dipping tanks in many rural areas has shifted the financial and logistical burden of tick control to individual households, often resulting in irregular acaricide use and practices that may promote resistance. These constraints highlight the importance of understanding tick infestation not only as a biological phenomenon but also as a product of governance, infrastructure, and household-level decision-making.

Against this backdrop, the present study investigated the socio-economic and management-related determinants of tick infestation in communal cattle systems of the Sinthumule–Kutama area, Limpopo Province, South Africa. By focusing on household characteristics, grazing practices, tick control behaviors, dipping infrastructure, economic impacts, and zoonotic exposure indicators, this study aimed to generate evidence that informs integrated, context-appropriate tick management strategies. The findings are intended to support policy development and intervention planning that address livestock productivity, rural livelihoods, and public health risk mitigation within a One Health framework.

## 2 Materials and methods

### 2.1 Study area and production system context

This study was conducted in the Sinthumule–Kutama municipality, Vhembe District, Limpopo Province, South Africa. The area is predominantly rural and characterized by communal cattle production systems in which animals are owned individually but managed collectively on shared grazing land. Climatic conditions are subtropical, with warm, wet summers and dry winters, creating an ecological setting conducive to year-round tick persistence. Communal grazing, shared watering points, and limited biosecurity measures typify the production system and create a close interface between livestock, humans, and the environment, making the area suitable for investigation of tick-related risks.

### 2.2 Study design and conceptual framework

A cross-sectional, mixed-methods study design was employed to investigate herd management practices, tick control strategies, and associated risks at household and community levels. The study was conceptually grounded in a One Health framework, integrating animal health, human exposure, and environmental management factors. Quantitative household survey data were linked to herd-level tick infestation metrics, focusing specifically on socio-epidemiological, management, and infrastructural factors associated with persistent tick exposure.

### 2.3 Household selection and study population

The study population comprised communal cattle-owning households across 17 villages in the study area. Village livestock registers were used as sampling frames. Within each village, approximately half of all cattle-owning households were randomly selected using computer-generated random numbers. Households were eligible if they owned at least one head of cattle and provided informed consent. A total of 168 households were enrolled, ensuring adequate representation across villages and sufficient statistical power to assess management and practice-related factors influencing tick exposure.

### 2.4 Questionnaire design and data collection

Data were collected using a structured questionnaire administered through face-to-face interviews with household heads or primary livestock caretakers. The questionnaire was developed based on previous studies on tick control and livestock management in communal systems and was pretested in two non-study villages to ensure clarity, cultural appropriateness, and reliability. Interviews were conducted in indigenous language, Tshivenda, by trained local field assistants and later translated into English language for analysis.

The questionnaire captured information on household demographics, income sources, herd size and composition, grazing systems, tick control practices, frequency and type of acaricide use, access to veterinary services, and perceptions of tick-related risks. Specific emphasis was placed on farmer knowledge of ticks and tick-borne diseases, including perceived risks to human health. The knowledge was operationalized as the ability to correctly name or describe at least one tick-borne disease and answer 2 of the 4 other propping questions. Data on decision-making related to tick control, constraints to effective implementation, and reliance on traditional or informal practices were also recorded to assess knowledge–practice gaps.

### 2.5 Assessment of tick control infrastructure

All village dipping facilities serving the study communities were physically inspected during the study period. Each facility was assessed for operational status, frequency of use, accessibility to households, availability of acaricides, and evidence of recent maintenance. Information obtained from household interviews regarding dipping practices was cross-validated against observations at dipping sites and discussions with community animal health workers. This approach allowed triangulation of reported practices with infrastructural realities and improved the reliability of management-related findings.

### 2.6 Tick burden

For analytical purposes, herd-level tick burden was defined as mean tick count per animal derived from standardized partial-body counts of 10 animals per herd (randomly selected or all animals if ≤ 5), following field-based tick assessments conducted during animal examinations. A minimum of 5 animals per herd were examined. For herds < 5, all cattle in the herd were assessed. Tick counts were recorded using a standardized partial-body counting approach focusing on anatomically defined predilection sites, and individual animal counts were summed to obtain herd-level tick counts for each household as previously reported (30, 31).

### 2.7 Data management and statistical analysis

Survey data were entered into Microsoft Excel and checked for completeness and consistency before analysis. Descriptive statistics were used to summarize household demographics, income levels, herd sizes, grazing systems, and tick control practices. Associations between management variables and aggregated tick burden were explored using chi-square tests and non-parametric comparisons as deemed appropriate. To account for clustering at village level, generalized linear mixed-effects models (GLMM) with a negative binomial distribution and log link function were applied with village included as a random effect. Tick counts were modeled as over dispersed count data using a negative binomial GLMM. Model fit was assessed using the Akaike Information Criterion (AIC), residual diagnostics, and overdispersion tests. Multicollinearity was evaluated using the Variance Inflation Factor (VIF). Statistical analyses were performed using SPSS version 25, with significance set at *p* < 0.05.

### 2.8 Ethical considerations

Ethical approval for the study was obtained from the University of South Africa College of Agriculture and Environmental Sciences Research Ethics Committee (Ref: 2020/CAES_HREC/014). Written informed consent was obtained from all participating households. Confidentiality of respondents was maintained, and participation was entirely voluntary. All field activities were conducted in accordance with accepted ethical standards for research involving human participants and livestock-owning communities.

## 3 Results

### 3.1 Socio-demographic characteristics of cattle-owning households

A total of 168 communal cattle-owning households across 17 villages participated in the study. The majority of respondents were male household heads (63.1%), while females accounted for 36.9%. Most respondents were older adults, with 58.9% aged ≥50 years, reflecting the aging demographic profile of communal livestock keepers. Formal education levels were generally low; 47.6% had only primary education, and 21.4% had no formal education.

Household income was predominantly low, with 76.2% earning less than ZAR 5,000 (≈USD 310) per month, largely derived from social grants, subsistence farming, or informal employment. Cattle ownership was strongly associated with livelihood security rather than commercial production, with herds being savings assets, draft power, and socio-cultural capital. Household demographic and socio-economic characteristics are summarized in Table 1.

**Table 1:** Socio-demographic and economic characteristics of communal cattle-owning households (*n* = 168) in the Sinthumule–Kutama area, Limpopo Province, South Africa.

S/n	Variable	Category	Frequency (*n*)	Percentage (%)
1	Gender	Male	106	63.1
		Female	62	36.9
2	Age group (years)	< 40	28	16.7
		40–49	41	24.4
		≥50	99	58.9
3	Education level	No formal education	36	21.4
		Primary	80	47.6
		Secondary	42	25.0
		Tertiary	10	6.0
4	Monthly household income (ZAR)	< 2,500	71	42.3
		2,500–4,999	57	33.9
		≥5,000	40	23.8
5	Primary livelihood source	Social grants	89	53.0
		Farming	41	24.4
		Informal employment	26	15.5
		Formal employment	12	7.1

1 USD = ZAR 16.2.

### 3.2 Herd size, composition, and grazing systems

Herd sizes ranged from 1 to 68 cattle per household, with a median herd size of 7 animals. Small herds (≤10 cattle) constituted 61.9% of households, while only 12.5% owned more than 30 cattle. Grazing systems were predominantly communal, with 82.7% of households grazing cattle on shared rangelands, often characterized by bush encroachment and proximity to wetlands or seasonal streams. A smaller proportion relied on roadside grazing (11.3%) or a combination of communal and privately fenced land (6.0%).

Households practicing exclusive communal grazing had significantly higher aggregated herd-level tick burdens compared to those with partial access to private land (*p* < 0.05). Herd structure and the spatial distribution of grazing systems across villages are presented in Table 2.

**Table 2:** Herd size distribution, herd composition, and grazing systems among communal cattle-owning households (*n* = 168) in South Africa.

S/n	Variable	Category	Frequency (*n*)	Percentage (%)
1	Herd size	≤10 cattle	104	61.9
		11–30 cattle	43	25.6
		>30 cattle	21	12.5
2	Herd composition	Mixed age and sex	149	88.7
		Breeding females only	19	11.3
3	Grazing system	Communal rangeland	139	82.7
		Roadside grazing	19	11.3
		Mixed communal/private	10	6.0
4	Night kraaling	Yes	156	92.9
		No	12	7.1

### 3.3 Tick control practices and acaricide use

Tick control practices varied widely across households. Although 95.8% of respondents reported awareness that ticks cause disease in cattle, only 54.8% reported regular tick control, defined as at least once every two weeks during peak tick seasons. Pour-on acaricides were the most commonly used method (61.3%), followed by hand spraying (51.2%). Only 18.5% of households reported consistent use of plunge dipping.

Acaricide rotation was rarely practiced, 72.6% of respondents reported using the same product repeatedly, often based on availability rather than veterinary recommendation. Financial constraints were the most frequently cited reason for irregular control (68.4%), followed by lack of access to dipping facilities (57.1%). Tick control practices and acaricide use patterns are detailed in Table 3.

**Table 3:** Tick control practices and acaricide use among communal cattle-owning households (*n* = 168) in the Sinthumule–Kutama municipality of South Africa.

S/n	Variable	Category	Frequency (*n*)	Percentage (%)
1	Awareness that ticks cause disease	Yes	161	95.8
		No	7	4.2
2	Regular tick control practiced	Yes	92	54.8
		No	76	45.2
3	Tick control method used^*^	Pour-on	103	61.3
		Hand spraying	86	51.2
		Plunge dipping	31	18.5
4	Acaricide rotation practiced	Yes	46	27.4
		No	122	72.6
5	Main constraint to tick control	Financial limitation	115	68.4
		Lack of dipping access	96	57.1
		Lack of acaricides	61	36.3

^*^Multiple responses allowed.

### 3.4 Functionality and accessibility of dipping infrastructure

Dipping infrastructure status and accessibility are summarized in Table 4. Inspection of village dipping tanks revealed widespread infrastructural challenges. Of the dipping facilities serving the 17 villages, 94.6% were either non-functional or partially functional at the time of the study. Common issues included cracked tanks, lack of acaricide supply, irregular water availability, and absence of maintenance schedules. Even where tanks existed, only 41.7% of households lived within 5 km of a functional facility, limiting routine access. Households without access to functional dipping tanks had significantly higher aggregated tick burdens at herd level compared to those with access (*p* < 0.01).

**Table 4:** Functional status and accessibility of dipping infrastructure serving communal cattle-owning households (*n* = 168) in South Africa.

S/n	Variable	Category	Frequency (*n*)	Percentage (%)
1	Dipping tank status	Functional	9	5.4
		Partially functional	48	28.6
		Non-functional	111	66.0
2	Distance to nearest functional tank	≤5 km	70	41.7
		>5 km	98	58.3
3	Frequency of community dipping	Weekly	18	10.7
		Irregular	62	36.9
		Not conducted	88	52.4
4	Acaricide supply at tanks	Adequate	22	13.1
		Inadequate/none	146	86.9

### 3.5 Farmer's knowledge of tick-borne and zoonotic diseases

Although general awareness of ticks was high, only 3.6% of respondents could correctly name at least one tick-borne disease, such as heartwater or babesiosis. Knowledge of zoonotic tick-borne diseases was particularly limited, as less than 10% recognized that ticks could transmit diseases to humans. Despite this, 78.0% reported frequent direct contact with ticks, either during herding, milking, or manual tick removal.

Knowledge and perception indicators are presented in Table 5. Use of personal protective measures was rare, with only 6.5% reporting glove use during tick removal. These findings reveal a substantial knowledge–practice gap of One Health importance, where awareness of ticks does not translate into effective risk mitigation.

**Table 5:** Farmer knowledge, perceptions, and practices related to tick-borne and zoonotic diseases among South African communal cattle farmers (*n* = 168).

S/n	Variable	Category	Frequency (*n*)	Percentage (%)
1	Can name a tick-borne disease	Yes	6	3.6
		No	162	96.4
2	Aware ticks can infect humans	Yes	15	8.9
		No	153	91.1
3	Frequent direct contact with ticks	Yes	131	78.0
		No	37	22.0
4	Uses protective measures	Yes	11	6.5
		No	157	93.5
5	Manually removes ticks	Yes	124	73.8
		No	44	26.2

### 3.6 Knowledge–practice gaps and determinants of ineffective tick control

Cross-tabulation of farmer knowledge and reported practices revealed that 42.9% of households aware of tick risks did not implement regular control measures. Households with lower income, greater distance to dipping facilities, and reliance on communal grazing were significantly more likely to exhibit ineffective tick control practices (*p* < 0.05).

Mixed-effects models identified lack of functional dipping infrastructure, low household income, and exclusive communal grazing as significant predictors of high herd-level tick burden, after accounting for village-level clustering. These associations are summarized in Table 6.

**Table 6:** Factors associated with ineffective tick control and high herd-level tick burden among communal cattle-owning households (mixed-effects analysis).

S/n	Factors	Adjusted effect (β)	SE	*P*-value
1	Lack of functional dipping tank	+0.41	0.09	< 0.001
2	Low household income (< ZAR 5,000)	+0.29	0.07	< 0.001
3	Exclusive communal grazing	+0.25	0.08	0.002
4	Irregular acaricide use	+0.33	0.06	< 0.001
5	Village-level clustering (ICC)	0.38	—	—

1 USD = ZAR 16.2; ICC, intra-class correlation coefficient; SE, standard error.

### 3.7 Economic implications of tick infestation

Farmers reported multiple economic impacts attributed to tick infestation, including poor body condition, reduced draft power, hide damage, and increased expenditure on acaricides. 61.9% of respondents reported spending more than ZAR 1,000 (USD 62) annually on tick control, representing a substantial proportion of household income. Losses associated with reduced market value of cattle and delayed sales were frequently cited, particularly during peak infestation periods. Households with higher reported tick burdens were significantly more likely to report negative economic outcomes (*p* < 0.01). Economic impacts associated with tick infestation are summarized in Table 7.

**Table 7:** Reported economic impacts associated with tick infestation among communal cattle-owning households (*n* = 168) in South Africa.

S/*n*	Economic impact	Frequency (*n*)	Percentage (%)
1	Reduced body condition	112	66.7
2	Reduced draft power	98	58.3
3	Hide damage	87	51.8
4	Increased acaricide expenditure	104	61.9
5	Delayed cattle sales	69	41.1
6	Annual tick control cost > ZAR 1,000	104	61.9

1 USD = ZAR 16.2

## 4 Discussion

This study provides a comprehensive socio-ecological and management-focused assessment of tick infestation in communal cattle systems of the study area, revealing that persistent tick burdens are primarily driven by structural, socio-economic, and management factors rather than host biology alone. Unlike animal-level analyses reported elsewhere (32, 33), the present findings demonstrate that ineffective tick control in communal systems is deeply embedded in poverty, infrastructural decay, and knowledge–practice gaps. These factors collectively sustain high exposure risks for livestock, humans, and the environment. These findings reinforce the necessity of reframing tick control from a purely veterinary intervention to a One Health challenge requiring coordinated multi-sectorial responses at the human-animal-environment interphase.

The dominance of communal grazing systems and small herd ownership observed in this study is consistent with reports from other parts of sub-Saharan Africa, and South Africa in particular (34), where communal rangelands facilitate continuous tick–host–environment interactions. High stocking densities, uncontrolled animal movement, and shared grazing lands create ideal ecological conditions for tick survival and dispersal, particularly for multi-host species. Comparable study from Zambia and South Africa have similarly demonstrated higher tick infestation levels in communal systems compared to commercial farms, attributing this to limited pasture management and absence of rotational grazing (31, 32, 35). These ecological realities underscore why communal cattle systems often act as reservoir landscapes for tick populations and tick-borne pathogens.

One of the most critical findings of this study is the widespread dysfunction of dipping infrastructure. Over 90% of dipping tanks were non-functional or partially functional, severely undermining community-level tick control. Dipping tanks have historically served as the backbone of tick and tick-borne disease control in southern Africa, and their collapse represents not merely a technical failure but a systemic governance and service-delivery breakdown in animal agriculture. Similar infrastructural decline has been reported in communal areas of Burkina Faso and Zambia, where withdrawal of state support and inconsistent acaricide supply have led to resurgence of tick populations and associated diseases (36, 37). The strong association between lack of functional dipping facilities and elevated herd-level tick burden in this study highlights infrastructure as a determinant of animal health inequality.

Acaricide use patterns observed among households raise additional concerns regarding sustainability and resistance development. The widespread reliance on repeated use of single acaricide formulations without rotation, often applied at irregular intervals, mirrors patterns reported across other African countries, and is a recognized possible driver of acaricide resistance in most tick species (38–40). While resistance testing was beyond the scope of this study, the documented practices strongly suggest a high risk of emerging or established resistance, which could further compromise tick control efforts. From a One Health perspective, inappropriate acaricide use also poses risks of environmental contamination and human exposure, particularly in settings where application occurs without the use of protective equipment.

The study further reveals a profound gap between awareness of ticks and practical knowledge of tick-borne diseases. Although nearly all respondents recognized ticks as harmful to cattle, very few could name specific diseases or understood their zoonotic potential. This disconnect reflects limitations in extension services that prioritize product use over disease literacy. The high frequency of manual tick removal and direct contact without protective measures observed in this study substantially increases the risk of human exposure to zoonotic pathogens, including Rickettsia and other tick-borne agents of zoonotic and public health significance (41–43). These findings reinforce the need to integrate human health messaging into veterinary extension programs.

The economic consequences of tick infestation documented in this study are substantial and disproportionately affect already vulnerable households. Reported losses through reduced draft power, poor body condition, hide damage, and increased expenditure on acaricides represent both direct and opportunity costs that undermine livelihood resilience. For households dependent on cattle for plowing, bride wealth, or emergency income, these impacts translate into broader food security and socio-cultural consequences. Similar economic where tick infestation was linked to significant reductions in household income and agricultural productivity has been reported (20). Importantly, the high proportion of households spending more than ZAR 1,000 (USD 62) annually on tick control highlights the regressive nature of tick-associated costs in low-income systems.

From One Health perspective, the findings underscore the interconnectedness of animal health, human exposure, and environmental conditions in communal settings. Shared living spaces between humans and cattle, combined with limited biosecurity and high tick abundance, create conditions conducive to cross-species pathogen transmission (44–46). While clinical human tick-borne disease cases may be underreported, the exposure indicators identified in this study suggest a silent but persistent zoonotic risk. Addressing such risks requires breaking down traditional sectorial silos and embracing integrated One Health surveillance and intervention strategies.

The One Health implications of this study extend beyond local tick control to broader questions of policy and governance. The collapse of dipping infrastructure, limited veterinary outreach, and absence of coordinated human health engagement reflect systemic challenges in rural service delivery. Re-establishing functional dipping systems alone will be insufficient unless accompanied by community engagement, sustainable financing mechanisms, and inter-sectorial collaboration. Successful examples from Easter Cape Province, South Africa demonstrate that community-managed dipping schemes, supported by government and private stakeholders, can restore effective tick control while fostering ownership and compliance (47).

Finally, this study contributes to the growing body of evidence that effective tick management in communal systems must be context-sensitive, socially informed, and One Health-oriented. Interventions that fail to account for socio-economic constraints, cultural practices, and institutional realities are unlikely to achieve lasting impact. Therefore, this study provides a framework for reimagining tick control as a public good rather than a private burden borne by impoverished farmers.

## 5 Conclusion

Persistent tick infestation in communal cattle systems in the study area reflects the combined effects of socioeconomic limitations, deteriorating dipping infrastructure, suboptimal tick control practices, and inadequate disease literacy. These interconnected factors undermine livestock productivity and welfare while creating conditions for increased human exposure to ticks. Addressing this challenge requires coordinated reinvestment in accessible dipping services, strengthened community-based veterinary extension, and systematic integration of zoonotic risk communication and One Health surveillance into routine animal health programs.

### 5.1 Limitation of the study

Potential biases include recall bias (self-reported practices), social desirability bias, and possible selection bias due to reliance on livestock registers. These biases were reasonably mitigated through triangulation with field observations. Although this study did not assess clinical human tick-borne disease outcomes, the high frequency of direct human–tick contact, manual tick removal, and minimal use of protective measures represent plausible exposure pathways. These findings therefore indicate potential public health risk rather than disease burden, underscoring the need for integrated One Health surveillance and risk communication.

## Data Availability

The raw data supporting the conclusions of this article will be made available by the authors, without undue reservation.
